# Olive Flounder By-Product Prozyme2000P Hydrolysate Ameliorates Age-Related Kidney Decline by Inhibiting Ferroptosis

**DOI:** 10.3390/ijms25094668

**Published:** 2024-04-25

**Authors:** Myeongjoo Son, You-Jin Jeon, Bomi Ryu, Dae Yu Kim

**Affiliations:** 1 Department of Anatomy & Cell Biology, School of Medicine, Kangwon National University, Chuncheon 24341, Republic of Korea; mjson@kangwon.ac.kr; 2 Department of Marine Life Sciences, Jeju National University, Jeju 63243, Republic of Korea; youjinj@jejunu.ac.kr; 3 Marine Science Institute, Jeju National University, Jeju 63333, Republic of Korea; 4 Major of Food Science and Nutrition, Pukyong National University, Busan 48513, Republic of Korea; 5 Inha Research Institute for Aerospace Medicine, Inha University, Incheon 22212, Republic of Korea; 6 Department of Electrical and Computer Engineering, College of Engineering, Inha University, Incheon 22212, Republic of Korea; 7 Center for Sensor Systems, Inha University, Incheon 22212, Republic of Korea

**Keywords:** aging, olive flounder by-product Prozyme2000P (OFBP), ferroptosis, nephropathy, cell senescence

## Abstract

This study explores olive flounder by-product Prozyme2000P (OFBP) hydrolysate as a potential treatment for age-related kidney decline. Ferroptosis, a form of cell death linked to iron overload and oxidative stress, is increasingly implicated in aging kidneys. We investigated whether OFBP could inhibit ferroptosis and improve kidney health. Using TCMK-1 cells, we found that OFBP treatment protected cells from ferroptosis induced by sodium iodate (SI). OFBP also preserved the mitochondria health and influenced molecules involved in ferroptosis regulation. In aging mice, oral administration of OFBP significantly improved kidney health markers. Microscopic examination revealed reduced thickening and scarring in the kidney’s filtering units, a hallmark of aging. These findings suggest that OFBP hydrolysate may be a promising therapeutic candidate for age-related kidney decline. By inhibiting ferroptosis, OFBP treatment appears to improve both cellular and structural markers of kidney health. Further research is needed to understand how OFBP works fully and test its effectiveness in more complex models.

## 1. Introduction

Ferroptosis, a distinct form of regulated cell death, differs from apoptosis, necrosis, and autophagy [1], and ferroptosis is a regulated form of cell death characterized by iron accumulation and lipid peroxidation [1]. In addition, ferroptosis has also been linked to the regulation of oxidative stress and inflammatory responses. It plays key roles in various diseases by connecting oxidative stress and inflammation, highlighting its importance beyond merely being a cell death mechanism [2]. Oxidative stress arises from an imbalance between reactive oxygen species (ROS) production and the body’s ability to detoxify these intermediates or repair the resulting damage. In the aging kidney, oxidative stress significantly contributes to cellular damage, inflammation, and a decline in renal function [3,4].

The aging kidney is characterized by a progressive decrease in its functional capacity. This decline, termed renal senescence, is attributed to a multitude of structural and functional changes [3,5]. There are some cellular and molecular alterations. (i) Nephron loss: a hallmark feature of renal aging is the gradual decline in the number of nephrons, the kidney’s functional units. This reduction is primarily due to age-related apoptosis and a diminished capacity for nephron renewal. (ii) Hemodynamic alterations: renal blood flow and glomerular filtration rate (GFR) progressively decline with age. This can be attributed to factors such as stiffening of the renal vasculature and a decline in cardiac output. (iii) Glomerular damage: the delicate filtration units of the kidney, the glomeruli, become increasingly sclerotic (scarred) with age. This process, termed glomerulosclerosis, hinders their ability to filter waste products from the blood effectively. (iv) Tubulointerstitial fibrosis: the supporting tissue within the kidney, the tubulointerstitium, undergoes progressive fibrosis with age. This disrupts the normal architecture of the kidney and further compromises its function [3,5]. These cellular and molecular alterations collectively contribute to the decline in kidney function observed in elderly individuals.

Iron accumulation in the kidney, particularly in chronic kidney disease (CKD), can initiate oxidative damage through Fenton-mediated reactions, contributing to renal injury [6]. Iron homeostasis is maintained by mechanisms such as hepcidin and iron regulatory proteins (IRPs), which regulate iron uptake and export. In CKD, dysregulation of these mechanisms leads to iron deposition, which is a key factor in inducing ferroptosis [6]. Additionally, lipid metabolism, especially the peroxidation of polyunsaturated fatty acids (PUFAs), plays a critical role in ferroptosis. Enzymes like acyl-CoA synthetase long-chain family member 4 (ACSL4) and glutathione peroxidase 4 (GPX4) are central to the regulation of lipid peroxidation and ferroptosis [6,7].

Natural products have emerged as a promising source of novel ferroptosis inhibitors, offering several advantages over synthetic drugs. For example, baicalein, a flavonoid extracted from Scutellaria species, has been shown to potently inhibit erastin-induced ferroptosis in pancreatic cancer cells [8]. Baicalein’s mechanism of action involves limiting ferrous iron production, glutathione depletion, and lipid peroxidation, while also suppressing the degradation of GPX4, a key ferroptosis suppressor. A comprehensive review identified over 60 natural products with anti-ferroptosis activity, highlighting their therapeutic potential [9].

In this study, we aimed to investigate the potential of natural compounds in inhibiting ferroptosis, using olive flounder (OF, Paralichthys olivaceus) as the research subject. The OF species is highly valued in Eastern Asia due to its fast growth and high disease resistance, making it an important species for aquaculture [10]. An extract from OF was selected to evaluate its efficacy in inhibiting ferroptosis in the aging kidney, with in vitro and in vivo models being used for the evaluation.

## 2. Results

### 2.1. OFBP Treatment Effectively Mitigates Cell Death in Sodium Iodate (SI)-Treated TCMK-1 Cells

To further investigate the protective effects of OFBP against ferroptosis, we established a sodium iodate (SI)-induced ferroptosis model in vitro [11]. Sodium iodate (NaIO_3_) is a potent oxidizing agent known to induce cellular senescence in vitro through oxidative stress, NAD+ depletion, DNA damage, and mitochondrial dysfunction [12]. To validate the effectiveness of OFBP treatment in reducing ferroptosis, we prepared OFBP materials. Subsequently, the peptide sequence GASGERGEVGPA was validated and analyzed using liquid chromatography-tandem mass spectrometry (LC-MS/MS). The LC-MS/MS spectrum of GASGERGEVGPA showed peaks at 543.76322+ m/z, and the molecular mass and amino sequences determined are presented in Supplementary Figure S1. 

**Figure 1:**
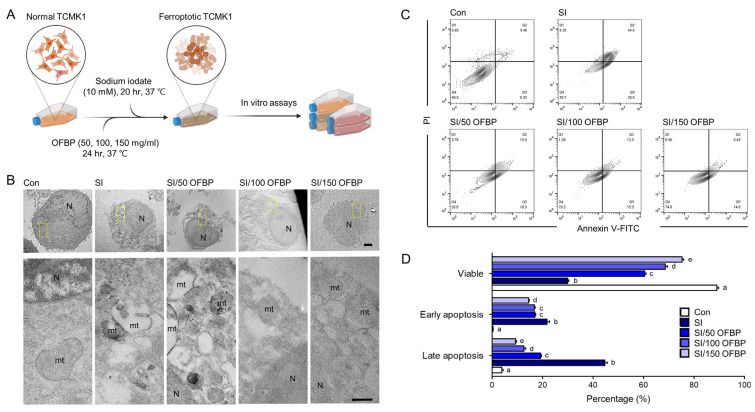
Attenuation of cell death in sodium iodate (SI)-treated TCMK-1 cells by OFBP. (**A**) TCMK-1 cells were initially treated with various concentrations of OFBP (50, 100, and 150 mg/mL) for 24 h, followed by a 20 h treatment with 10 mM sodium iodate (SI). Subsequently, several in vitro assays were performed to assess cellular responses. (BioRender, https://www.biorender.com/ (accessed on 24 April 2024) was used to generate the figure). (**B**) Representative transmission electron microscopy (TEM) images (scale bar: upper panel, 2 µm; lower panel, 500 µm) depicting notable ultracellular structure changes in TCMK-1 following treatment with SI, with or without OFBP treatment. (**C**,**D**) Cell viability was assessed using Annexin V/PI-based Cell Apoptosis Detection assays. (**D**) The graph represents the mean ± SEM of viable, early apoptotic, and late apoptotic cell numbers. Distinct letters above the columns indicate statistically significant differences between groups (*p* < 0.05). FITC, fluorescein isothiocyanate; mt, mitochondria; N, nucleus; OFBP, olive flounder by-product Prozyme 2000P; PI, propidium iodide; SI, sodium iodate; TEM, transmission electron microscope.

Next, we established a model system that allows us to evaluate the ability of OFBP to prevent ferroptosis in kidney tubular cells. Before treating the cells with 10 mM SI for 20 h, we exposed them to three different concentrations (50, 100, and 150 mg/mL) of OFBP for 24 h (Figure 1A). Following the OFBP treatment, we conducted in vitro assays. Mouse kidney tubular epithelial cells (TCMK-1 cells) exhibited significant ultrastructural changes after SI treatment, including alterations in the plasma membrane, mitochondrial shrinkage, and reduction or disappearance of mitochondrial cristae (Figure 1B). Apoptotic phenotype was also substantially increased by SI treatment (Figure 1C,D). The Annexin V assay revealed that 44.7% ± 1.6% of cells exhibited a late apoptotic phenotype (Q2) and 21.7 ± 1.7% showed an early apoptotic phenotype (Q3) after 20 h of SI treatment. However, Annexin V-FITC/PI cell apoptosis detection analysis demonstrated that treatment with OFBP at concentrations of 50, 100, and 150 mg/mL (SI/50 OFBP, SI/100 OFBP, and SI/150 OFBP groups) restored cell viability and reduced cell death (Figure 1C,D). Interestingly, the SI/150 OFBP group exhibited the highest percentage (75.3 ± 1.3%) of viable cells compared to all other treatment groups.

### 2.2. OFBP Treatment Is Effective in Reducing Ferroptosis in TCMK-1 Cells Treated with SI

**Figure 2:**
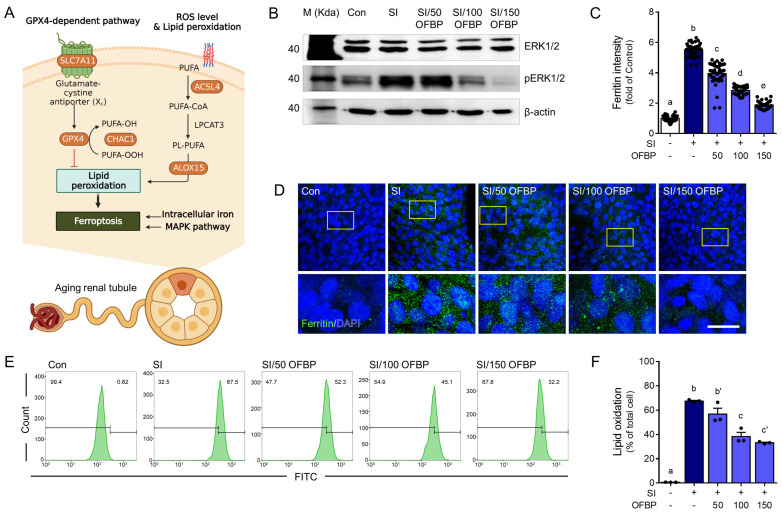
Attenuating ferroptosis-related pathway in sodium iodate (SI)-treated TCMK-1 cells by OFBP. (**A**) The in vitro experimental protocol is depicted in a flow diagram, illustrating the cultivation of TCMK-1 cells. The cells were initially treated with three concentrations of OFBP (50, 100, and 150 mg/mL) for 24 h, followed by a 20 h treatment with 10 mM sodium iodate (SI). Subsequently, various in vitro assays were performed to assess cellular responses. (BioRender https://www.biorender.com/ (accessed on 13 April 2024) was used to generate the figure.) (**B**) A representative Western blot showed protein expression levels of ERK1/2 and pERK1/2 in TCMK-1 cells treated with SI, with or without OFBP pretreatment. (**C**,**D**). (**C**) The graph and (**D**) representative fluorescence images depicting ferritin protein expression in TCMK-1 cells treated with SI, with or without OFBP pretreatment. The image in the yellow box is enlarged below from above the images. (**E**,**F**) The graphs show changes in lipid peroxidation levels in TCMK-1 cells treated with SI, with or without OFBP pretreatment. The results are expressed as the mean ± SEM. Distinct letters above the columns indicate statistically significant differences between groups (*p* < 0.05). Scale bar = 20 µm. Alox15, Arachidonate 15-Lipoxygenase; Chac1, CHAC Glutathione Specific Gamma-Glutamylcyclotransferase 1; ERK1/2; Extracellular signal-regulated kinase 1/2; Gpx4, Glutathione peroxidase 4; MDA, Malondialdehyde; OFBP, olive flounder by-product Prozyme 2000P; Slc7a11, Solute carrier family 7 member 11. pERK1/2; Phosphorylated extracellular signal-regulated kinase ½; OFBP, olive flounder by-product Prozyme 2000P; SI, Sodium iodate.

This result investigates the influence of SI treatment, alone or in combination with OFBP treatment, on ferroptosis induction in TCMK-1 cells. Ferroptosis, a form of iron-dependent cell death characterized by ROS accumulation and lipid peroxidation, is meticulously regulated by genes and signaling pathways governing iron homeostasis, lipid metabolism, and oxidative stress response [13,14]. To elucidate the mechanisms underlying SI’s and/or OFBP’s influence on ferroptosis, we evaluated Ferritin expression, a well-established marker of cellular iron storage, alongside lipid oxidation and the protein kinase extracellular signal-regulated kinase (ERK) signaling pathway, both of which are known to contribute to ferroptosis execution (Figure 2A).

SI treatment significantly increased ERK1/2 phosphorylation compared to the control group (Figure 2B). Conversely, OFBP treatment effectively reduced this phosphorylation, except in the SI/50 OFBP group (Figure 2B). Consistent with the ERK1/2 data, ferritin protein expression, a marker of cellular iron storage, was elevated in SI-treated cells compared to controls. Notably, OFBP treatment reversed this effect, with decreasing ferritin levels observed in the 50, 100, and 150 mg/mL OFBP groups (Figure 2B–D). These findings align with previous reports demonstrating that SI treatment upregulates ferritin light chain (FTL) mRNA and protein levels in human retinal pigment epithelium ARPE-19 cells [15]. 

Furthermore, FITC staining, a marker of lipid oxidation, revealed a significant increase in lipid-oxidized cells following SI treatment compared to the control group. Like the observed trends with ERK1/2 phosphorylation and ferritin expression, OFBP treatment significantly reduced the number of FITC-positive cells, except for the SI/50 OFBP group (Figure 2E,F). 

### 2.3. OFBP Administration Ameliorates Histological Characteristics in Aging Kidney 

**Figure 3:**
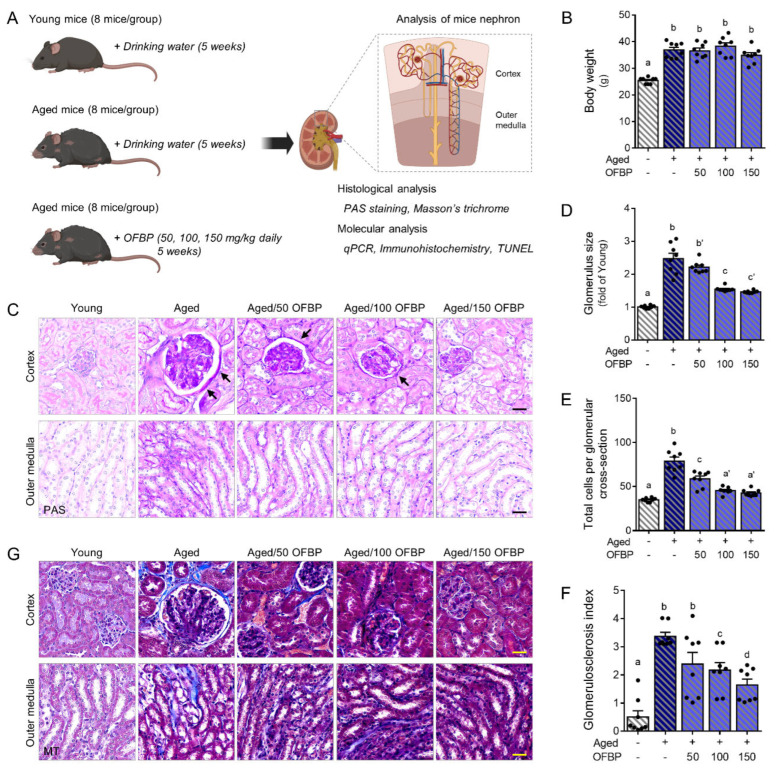
Effects of olive flounder by-product (OFBP) on kidney histology and function in young and aged mice. (**A**) Aged mice were randomly assigned to receive either drinking water or olive flounder by-product (OFBP) at three doses (50, 100, or 150 mg/kg/day) for 5 weeks. After treatment, several in vivo assays were performed to assess kidney function and cellular responses. (BioRender https://www.biorender.com/ (accessed on 13 April 2024) was used to generate the figure.) (**B**) The graph shows no significant differences in body weight among the mice treated with drinking water or various doses of OFBP for 5 weeks. (**C**–**G**) Representative images of kidney sections stained with (**C**) Periodic Acid-Schiff (PAS) and (**G**) Masson’s trichrome (MT), which are used to evaluate tissue morphology. The arrows indicate a positively stained basement membrane in the kidney. The (**D**) glomerulus size, (**E**) total cells per glomerular cross-section, and (**F**) glomerulosclerosis index were quantified from (**C**) PAS-stained images. (**G**) MT images show fibrosis (blue color) in the renal tissues. The scale bar represents 30 µm. The results are expressed as the mean ± SEM. Distinct letters displayed above the columns indicate significant differences between the groups (*p* < 0.05). MT, Masson’s trichrome; OFBP, olive flounder by-product Prozyme 2000P; PAS, Periodic Acid-Schiff.

Building upon our in vitro findings on OFBP’s potential to inhibit ferroptosis, we investigated its effects in an aged mouse model. We established a comprehensive analysis of age-related histological changes in the kidney. To evaluate OFBP’s efficacy, mice were administered oral doses of 50, 100, or 150 mg/kg/day for five weeks. Young and aged control mice received drinking water during the same period (Figure 3A).

Aging kidneys are characterized by distinct structural alterations, including glomerular hypertrophy (enlargement) and thickening of the glomerular basement membrane (GBM) [16]. Notably, OFBP treatment did not affect body weight changes (Figure 3B). Periodic Acid-Schiff (PAS) staining revealed a clear visualization of GBM (marked by arrows) and glomerular hypertrophy in aged mice. Compared to young controls, aged mice displayed significantly increased glomerular size and total cell number per glomerulus. These parameters were significantly reduced following OFBP administration (Figure 3C–E). 

Glomerulosclerosis, a hallmark of aging kidneys, refers to the hardening and scarring of glomeruli. The Glomerulosclerosis Index (GSI) serves as a quantitative measure of this process. Typically, GSI is elevated in aged kidneys, reflecting heightened glomerulosclerosis [17]. Consistent with this, aged mice exhibited a significantly higher GSI compared to young mice. However, OFBP administration significantly decreased GSI (Figure 3F). Furthermore, the fibrotic area within the renal cortex and outer medulla was considerably greater in aged mice compared to young controls. This fibrotic area was significantly diminished following OFBP treatment (Figure 3G).

### 2.4. OFBP Administration Decreased Cell Death in Aging Kidney

**Figure 4:**
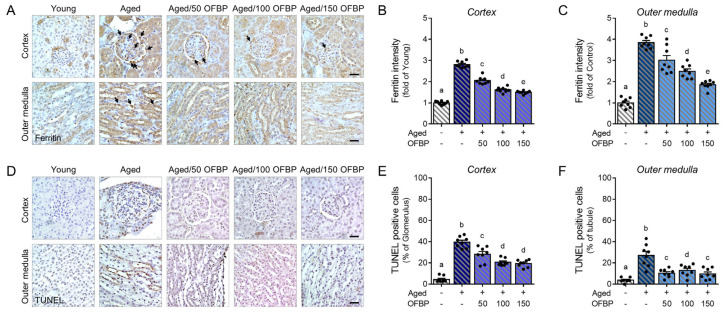
Attenuation of cell death in aged mice kidney by OFBP administration. (**A**) Representative images show ferritin protein expression (deep brown, arrows) in the renal cortex and outer medulla of mice kidneys. Scale bar: 30 μm (**B**,**C**) The graphs quantify ferritin intensity per renal tubule in the (**B**) cortex and (**C**) outer medulla. (**D**) Representative images from the TUNEL assay show apoptotic cells with brown punctate staining in the cortex and outer medulla of mice kidneys. Scale bar: 30 μm. (**E**,**F**) The graphs quantify the number of apoptotic cells detected by TUNEL assay in the (**E**) cortex and (**F**) outer medulla. Data are presented as mean ± SEM. Different letters above the columns indicate statistically significant differences between groups (*p* < 0.05). OFBP, olive flounder by-product Prozyme 2000P; TUNEL, Terminal deoxynucleotidyl transferase dUTP nick end labeling.

Consistent with previous report in aged animals, our study observed elevated ferritin protein expression in the kidneys of aged mice compared to young controls (Figure 4A–C) [18]. This finding aligns with the notion that ferritin levels increase during aging, potentially reflecting altered iron homeostasis and redox regulation. Notably, OFBP administration significantly decreased ferritin expression in both the renal cortex and outer medulla, suggesting a potential role for OFBP in modulating iron metabolism within the aging kidney.

Furthermore, we investigated apoptosis, a form of programmed cell death, using the Terminal deoxynucleotidyl transferase dUTP nick end labeling (TUNEL) assay. The number of TUNEL-positive cells, identified by their deep brown nuclei, was significantly increased in the kidneys of aged mice compared to young controls (Figure 4D–F). Interestingly, OFBP administration led to a significant decrease in TUNEL-positive cells.

These observations on ferritin expression and apoptosis suggest a potential link between OFBP’s protective effects and the regulation of ferroptosis in the aging kidney.

### 2.5. OFBP Administration Is Reduced Ferroptosis-Related Gene Expression in Aged Kidney

**Figure 5:**
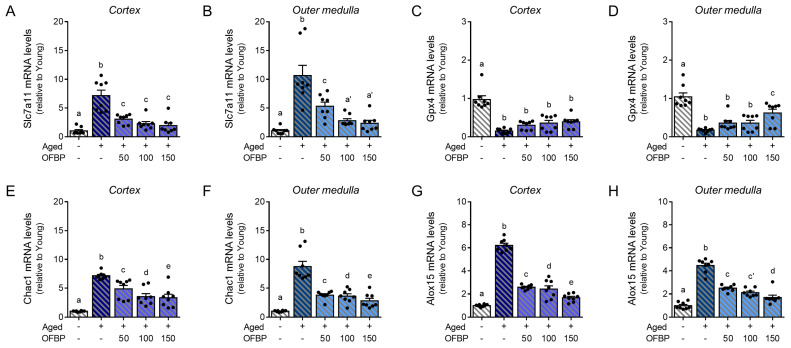
Effective mitigation of ferroptosis in aged mice kidney by OFBP administration (**A**–**H**) mRNA expression of ferroptosis-related genes (Slc7a11, Gpx4, Chac1, and Alox15) in the renal cortex (blue) and outer medulla (sky blue) of mice (8 mice/group) using RT-qPCR. Data are mean ± SEM. Different letters above the columns indicate statistically significant differences between groups (*p* < 0.05). Alox15, Arachidonate 15-Lipoxygenase; Chac1, CHAC Glutathione Specific Gamma-Glutamylcyclotransferase 1; Gpx4, Glutathione peroxidase 4; MDA, Malondialdehyde; OFBP, olive flounder by-product Prozyme 2000P; Slc7a11, Solute carrier family 7 member 11.

To elucidate the impact of aging on ferroptosis regulation, we employed RT-qPCR to evaluate the mRNA expression levels of key genes involved in the ferroptosis signaling pathway, including Solute Carrier Family 7 Member 11 (*Slc7a11*), *Gpx4,* ChaC Glutathione Specific Gamma-Glutamylcyclotransferase 1 *(Chac1*)*,* and Arachidonate 15-Lipoxygenase *(Alox15*). Kidney tissues from aged mice were dissected to isolate the cortex and outer medulla for independent analysis (Figure 5).

Compared to young mice, aged mice displayed significantly upregulated mRNA levels of *Slc7a11*, *Chac1*, and *Alox15* in both the cortex and outer medulla (Figure 5A,B,E–H). Conversely, *Gpx4* mRNA expression was downregulated in both regions of the aged kidney (Figure 5C,D). Interestingly, OFBP administration effectively restored the mRNA expression levels of all these genes to those observed in young mice. These findings suggest that OFBP may counteract the age-related dysregulation of the ferroptosis signaling pathway within the kidney. 

## 3. Discussion

This study investigates the protective effects of a novel compound, OFBP, against ferroptosis-mediated kidney damage in the context of aging. Ferroptosis, a distinct form of regulated cell death characterized by iron-dependent lipid peroxidation and mitochondrial dysfunction, is increasingly recognized as a potential contributor to various age-related pathologies [19,20,21]. Unlike other cell death processes, ferroptosis relies on specific metabolic pathways for lipid peroxidation and exhibits characteristic morphological changes in mitochondria, including shrinkage, increased membrane density, and a reduced or absent cristae structure [11,21,22]. 

Our findings demonstrate that OFBP treatment significantly mitigates ferroptosis in SI-induced TCMK-1 cells. This protective effect is evidenced by the preservation of mitochondrial morphology (reduced cristae loss and shrinkage) and a concomitant increase in viable cell numbers (Figure 1B–D). Additionally, OFBP treatment effectively reduces lipid oxidation, a hallmark characteristic of ferroptosis (Figure 2E,F).

Aging kidneys exhibit several characteristic microscopic structural changes, including thickening of the glomerular basement membrane (GBM), nephrosclerosis, accumulation of extracellular matrix, mesangial widening, and nephron hypertrophy [23,24]. The GBM is a critical component of the kidney’s filtration barrier, allowing for selective permeability. It retains essential proteins and cells in the bloodstream while preventing waste products and excess water from the urine [23,24]. PAS staining highlights the GBM with a deep pink color, and our results revealed an age-related increase in GBM thickness (Figure 3C, arrow). Thickening of the GBM can impair its filtering function, potentially leading to proteinuria (excess protein in the urine). The aging kidney experiences progressive accumulation of extracellular matrix proteins, such as collagen and fibronectin, leading to scarring and tissue stiffening (fibrosis). This process is a hallmark of renal aging and contributes to the decline in kidney function observed in older individuals. Senescent cells accumulate within the kidney and secrete pro-fibrotic factors that stimulate the deposition of extracellular matrix components, ultimately contributing to fibrosis. MT staining is typically used to evaluate fibrosis (Figure 3G). These histological hallmarks of kidney damage were attenuated in aged mice following OFBP administration. This renoprotective effect was confirmed by PAS and MT staining, demonstrating a decrease in the glomerular size and glomerulosclerosis index (GSI) in the OFBP-treated group compared to the control group (Figure 3C,D,F,G). 

Some studies suggest a multifaceted role for natural products in regulating ferroptosis, a form of iron-dependent cell death. These compounds exhibit diverse effects depending on the cellular context, with some acting as ferroptosis inhibitors and others potentially inducing it. Beyond Baicalein, curcumin, a polyphenol derived from Curcuma longa (turmeric), exemplifies this complexity. Studies have demonstrated its renoprotective properties in rhabdomyolysis, where it mitigates kidney damage and ferroptosis by reducing lipid peroxidation, possibly through activation of the Heme oxygenase-1 (HO-1) enzyme [25]. Conversely, curcumin has been shown to induce ferroptosis in breast cancer cells by promoting iron accumulation and HO-1 upregulation [26]. 

Epigallocatechin gallate (EGCG), a major polyphenol in green tea, has been identified as a promising ferroptosis inhibitor. Recent research suggests a novel role for EGCG in protecting pancreatic cells against erastin-induced ferroptosis. This protective effect is attributed to EGCG’s ability to chelate iron, prevent glutathione (GSH) depletion, and suppress lipid peroxidation [27]. Furthermore, EGCG administration has been shown to reduce oxidative stress and promote recovery in a spinal cord injury model, potentially by inhibiting ferroptosis [28]. 

Galangin, a flavonoid found in Alpinia officinarum and other plants, has also garnered attention for its potential to inhibit ferroptosis. Its mechanism of action likely involves reducing oxidative stress and maintaining cellular antioxidant capacity [29]. 

Sterubin, an active component isolated from the traditional medicinal herb Eriodictyon californicum, demonstrates promise as a neuroprotective agent by inhibiting ferroptosis in neuronal cell models. This study revealed sterubin’s ability to chelate iron, thereby preventing ferroptosis in glutamate-treated HT-22 mouse hippocampal neuronal cells [30]. This neuroprotective effect appears to be dose-dependent and mediated by increased GSH levels, decreased ROS production, and activation of the nuclear factor erythroid-2-related factor 2 (Nrf2) pathway [30]. The current study focused on establishing OFBP’s potential in an aged mouse model. We did not include an additional group receiving another ferroptosis inhibitor such as celastrol [31] for direct comparison. However, future studies will incorporate established ferroptosis inhibitors alongside OFBP to comparatively assess their efficacy in mitigating age-related kidney decline. 

We further explored the potential mechanism by which OFBP exerts its protective effects. Our data suggest that OFBP treatment modulates the expression of several key ferroptosis regulatory molecules in the kidney, including *Slc7a11*, *Gpx4*, *Chac1*, and *Alox15* (Figure 5). SLC7A11 and GPX4 are well-established ferroptosis suppressors, promoting glutathione synthesis and preventing lipid peroxidation, respectively [32,33,34]. CHAC1, although less frequently discussed, is involved in glutathione metabolism, potentially influencing cellular susceptibility to ferroptosis [35]. Finally, *Alox15* contributes to lipid peroxide production, and its regulation by OFBP suggests a potential mechanism for hindering ferroptosis progression (Figure 5). 

This study highlights the potential of OFBP as a novel therapeutic candidate for age-related kidney decline. By inhibiting ferroptosis, a critical pathway implicated in renal senescence, OFBP treatment demonstrably improves both cellular and histological markers of kidney health in vitro and in vivo models. Further investigation into the precise mechanisms underlying OFBP’s action and its efficacy in more complex pre-clinical models is warranted to advance its development as a potential therapeutic strategy for age-related kidney diseases.

## 4. Materials and Methods

### 4.1. Preparation of Olive Flounder By-Product Prozyme 2000P Hydrolysate (OFBP) in This Study 

The OFBP was prepared according to a previously described method [36] with slight modifications. Briefly, frozen OFB was thawed overnight at refrigerated temperatures (4 °C) and rinsed with water. The thawed OFB was then mixed with water and Prozyme 2000P enzyme at a specific ratio and a controlled pH range of 6.8–7.5. This mixture was incubated for 4 h to facilitate the breakdown of OFB by the enzyme complex within Prozyme 2000P. Following incubation, the mixture was heat-treated to inactivate the enzymes, filtered to remove solid particles, and concentrated using an evaporator. The concentrated extract was then freeze-dried for long-term storage.

### 4.2. UPLC-Q-TOF MS/MS Spectrum of Mass-Produced OFBP

The MS/MS analysis of OFBP was performed through ultra-high performance liquid chromatography-quadrupole time-of-flight (UPLC-Q-TOF) MS/MS at Proteinworks (Daejeon, Republic of Korea) using an Ultimate 3000 system (Dionex; Sunnyvale, CA, USA) and a Micro Q-TOF III mass spectrometer (Bruker Daltonics; Bremen, Germany). The column was Zorbax eclipse plus C18 (3.0 × 100 mm, 1.8 μm, Agilent, Hong Kong, China). The mobile phase consisted of A: H_2_O/0.1% formic acid and B: acetonitrile/0.1% formic acid with gradient method (flow rate 0.3 mL/min; 0–5 min, 98:2 *v*/*v*; 30 min, 80:20 *v*/*v*; 31–36 min, 2:98 *v*/*v*; 37–45 min, 98:2 *v*/*v*).

The presented chromatogram image represents the UV spectrum (red) and the base peak intensity mass chromatogram (blue) area of the mass-produced OFBP. Peptide sequencing was performed on the MS peak corresponding to *m*/*z* 543.7632 [M + 2H] at a retention time of 16.4 min, and the results confirmed a 12-amino acid sequence of GASGERGEVGPA (*m*/*z* 543.762+; 1085.5086 Da).

### 4.3. Sodium Iodate(SI)-Treated Ferroptosis Models (In Vitro)

This study employed mouse renal tubular epithelial cells (TCMK-1 cells) to establish in vitro models of sodium iodate (SI)-induced ferroptosis in renal tubular cells. TCMK-1 cells were derived from mouse kidney tissue and are commonly used as an in vitro model for studying renal physiology, pathology, and the effects of various substances on the kidneys. The cells were obtained from the American Type Culture Collection (ATCC, Manassas, VA, USA) and cultured in Dulbecco’s Modified Eagle’s Medium (DMEM; Welgene, Daegu, Republic of Korea) supplemented with 10% fetal bovine serum (FBS; Welgene) and 100 U/mL penicillin-streptomycin (Welgene). The cells were maintained in a humidified incubator with 5% CO_2_ at 37 °C.

To induce ferroptosis, TCMK-1 cells were treated with 10 mM sodium iodate (NaIO_3_) (Cat. # 71702; Sigma-Aldrich, St. Louis, MO, USA) and incubated in a humidified incubator under the same conditions.

### 4.4. Age-Related Kidney Change Models (In Vivo)

This animal study utilized male C57BL/6N mice. Kidneys were collected from all animals for further analysis. The mice were categorized into five groups (8 mice per group):

(1) Young group: 2-month-old mice were administered drinking water for 5 weeks.
(2) Aged group: 13-month-old mice were administered drinking water for 5 weeks.
(3) Aged group + olive flounder by-product Prozyme2000P hydrolysate (OFBP): 13-month-old mice orally were administered OFBP at the following doses for 5 weeks:
  (1) 50 mg/kg/day;
  (2) 100 mg/kg/day;
  (3) 150 mg/kg/day.

The study complied with the guidelines provided by the Institutional Animal Care and Use Committee of Pukyong National University (Busan, Republic of Korea) and received approval under the reference number PKNUIACUC-2023-04.

### 4.5. Annexin V-FITC/PI Cell Apoptosis Detection Analysis In Vitro

An Annexin V-FITC/PI Cell Apoptosis Detection Kit was used to quantify cell death following treatment with OFBP with or without SI. Briefly, confluent TCMK-1 cells were harvested by trypsinization and centrifugation, washed twice with cold-phosphate-buffered saline (PBS), and resuspended in 100 μL of Annexin V binding buffer. The cell suspension was then incubated with 5 μL of Annexin V-FITC and 5 μL of propidium iodide (PI) for 30 min in the dark on ice. Finally, 300 μL of binding buffer was added to each sample, and the cells were analyzed using a Calibur flow cytometer (BD Biosciences) for the determination of viable, apoptotic, and necrotic cell populations. The data were analyzed using BD CellQuest™ Pro Software (version 4.0.2).

### 4.6. Transmission Electron Microscopy (TEM) Examination

TCMK-1 cells were cultured on 10 cm² dishes and allowed to reach approximately 90% confluence. Subsequently, they were fixed with 2% glutaraldehyde (Sigma-Aldrich) for 4 h at room temperature to preserve their morphology. The cells were further fixed with 2% osmium tetroxide (OsO_4_) to enhance contrast for electron microscopy. After fixation, the cells were embedded in a resin for stabilization and sectioning. Ultrathin sections (around 60–90 nm thick) were obtained using a diamond knife. Finally, the prepared sections were observed under a Transmission Electron Microscope (TEM; Philips CM200) for high-resolution visualization of cellular ultrastructure. 

### 4.7. Lipid Peroxidation Analysis In Vitro

To assess lipid peroxidation, we first induced ferroptosis in TCMK-1 cells using sodium iodate (SI) with co-treatment of three different OFBP doses. Lipid peroxidation levels were then measured using the BODIPY™ 581/591 C11 fluorescent probe (Invitrogen, CA, USA). Briefly, cells were incubated with 5 μM BODIPY™ probe in fresh culture medium for 30 min at 37 °C. Subsequently, the cells were fixed with 4% paraformaldehyde (PFA) to preserve their morphology. Fluorescence intensity, indicative of lipid oxidation levels in TCMK-1 cells, was measured using a Calibur flow cytometer. The data were analyzed with BD CellQuest™ Pro Software.

### 4.8. Protein Isolation and Western Blotting

The TCMK-1 cells were lysed in RIPA buffer (ATTO, Tokyo, Japan) containing protease and phosphatase inhibitors. The lysate was centrifuged at 13,000× *g* for 20 min at 4 °C. The supernatant was collected and analyzed for protein concentration using a Pierce BCA protein assay kit (Thermo Fisher Scientific; Waltham, MA, USA). Western blotting was performed to verify protein expression. Briefly, 25 μg of total protein per lane was separated on a hand-cast 10% SDS-PAGE gel. Proteins were then transferred to PVDF membranes using semi-dry blotting (ATTO) at 500 mA for 40 min. To minimize non-specific binding, the PVDF membranes were blocked with 5% (*v*/*v*) skim milk in tris-buffered saline (TBS). Subsequently, membranes were incubated with primary antibodies against ERK, pERK (Cat. #bsm-52259R and bsm-54491R; Bioss, Beijing, China), and β-actin (Cat. #A5441; Sigma-Aldrich) at 4 °C for 48 h, followed by three washes with TBS containing 0.1% Tween-20 (TTBS). The membranes were then incubated with HRP-conjugated secondary antibodies at room temperature for 1 h. Finally, Western blotting signals were detected using the ChemiDoc XRS+ system (Bio-Rad Laboratories; Hercules, CA, USA).

### 4.9. Immunocytochemistry for Ferritin

TCMK-1 cells were seeded at 10,000 cells per well on 8-well chamber slides (SPL Life Sciences; Pocheon, Republic of Korea). Following washes with phosphate-buffered saline (PBS), cells were fixed with 4% paraformaldehyde (PFA) for 20 min. After fixation and PBS washes, the slides were blocked with normal animal serum for 1 h at room temperature. Subsequently, the cells were incubated with an anti-ferritin primary antibody, followed by PBS washes. Next, the cells were incubated with goat anti-rabbit IgG Alexa Fluor 488 secondary antibody (Cat. #A-11008, Thermo Fisher Scientific) for 1 h. Finally, nuclei were stained with DAPI, and the slides were mounted for visualization using an LSM 710 confocal microscope (Zeiss; Oberkochen, Germany) for the detection of ferritin expression and potential ferroptosis markers.

### 4.10. In Vivo Histology Analysis

Kidney tissues were fixed in 4% paraformaldehyde (PFA) for 2 days at 4 °C, followed by rinsing with distilled water. Subsequently, paraffin-embedded tissues were processed using a tissue processor and sectioned at 4 μm thickness. Sections were deparaffinized in xylene and rehydrated through an alcohol gradient for further analysis.

#### 4.10.1. Periodic Acid–Schiff (PAS) Stain

Periodic Acid–Schiff (PAS) staining, a technique for visualizing carbohydrates in tissues, is valuable in kidney research. It helps assess basement membrane integrity in kidney sections. Thickening, altered capillaries, and abnormal cells observed through PAS staining can indicate potential glomerular problems and impaired kidney function [37].

The slides were deparaffinized in xylene and rehydrated through an alcohol gradient. Subsequently, they were oxidized with 0.5% periodic acid solution for 5 min, followed by washes with water. The sections were then stained with Schiff reagent for 15 min and washed with lukewarm tap water. Finally, nuclei were counterstained with Mayer’s hematoxylin for 1 min and sections were washed with water. Stained sections were mounted with xylene based DPX mountant and visualized using light microscopy (Olympus; Tokyo, Japan). 

Glomerular damage was evaluated using a Glomerulosclerosis index (GSI, Grades 0–4) based on pre-defined criteria applied to randomly selected glomeruli in PAS-stained sections [37].

#### 4.10.2. Masson’s Trichrome (MT) Stain

The slides were re-fixed in Bouin’s solution for 24 h and rinsed with tap water. Tissue sections were stained with Weigert’s iron hematoxylin for 10 min, followed by Biebrich scarlet-acid fuchsin for 15 min. Differentiation was achieved using phosphomolybdic-phosphotungstic acid solution for 10 min. Finally, sections were stained with aniline blue solution for 3 min and washed with water. Stained sections were mounted with xylene-based DPX mountant and visualized using BX60 light microscopy. The MT staining differentiates various cellular components within the kidney tissue. In stained sections, renal tubules appear red, while collagen fibers are stained blue.

### 4.11. Immunohistochemistry for Ferritin

The deparaffinized tissue slides were subjected to antigen retrieval using 0.3% hydrogen peroxide for 10 min. After rinsing with PBS, sections were blocked with non-immune serum to reduce non-specific antibody binding. Subsequently, sections were incubated with anti-Ferritin antibody for 2 days. Following PBS washes, sections were incubated with biotinylated secondary anti-rabbit for 1 h and further incubated with an avidin-biotin complex (ABC) solution (Vector Laboratories, Burlingame, CA, USA) for signal amplification. After rinsing with PBS, immunoreactivity was visualized using 3,3′-diaminobenzidine (DAB, Sigma-Aldrish) as a chromogen for 1 min. Sections were then mounted with xylene-based DPX mountant and visualized using light microscopy (Olympus).

### 4.12. Terminal Deoxynucleotidyl Transferase-Mediated dUTP Nick end Labeling (TUNEL) In Vivo

Apoptosis was assessed using terminal deoxynucleotidyl transferase-mediated dUTP nick end labeling (TUNEL) according to the manufacturer’s protocol (Elabscience Biotechnology Co., Ltd., Wuhan, China). Deparaffinized kidney sections were stained using the TUNEL method to detect DNA fragmentation in apoptotic cells. Five sections from each group were randomly selected and analyzed using a light microscope (Olympus) to quantify TUNEL-positive cells (brown staining) of glomeruli in the cortex and outer medulla. ImageJ software (https://imagej.net/ij/) (National Institutes of Health; Bethesda, MD, USA) was used to quantify the number of TUNEL-positive cells.

### 4.13. RNA Isolation, cDNA Synthesis and Quantitative Real-Time PCR (RT-qPCR)

For gene expression analysis, total RNA was isolated from the mice’s renal cortex or outer medulla using RNAiso Plus (Cat. #9108; Takara Bio Inc., Tokyo, Japan). Following extraction, RNA was purified with isopropanol and ethanol washes. The quantity and quality of the isolated RNA were assessed using a NanoDrop 200 spectrophotometer (Thermo Fisher Scientific). Subsequently, cDNA synthesis was performed using a reverse transcription kit according to the manufacturer’s instructions. RT-qPCR was used to determine the mRNA expression levels of ferroptosis-related genes. Specific primers and 2X SYBR Green qPCR Master Mix (Cat. #QBLR-05; CellSafe, Seoul, Republic of Korea) were used in the reactions. β-actin (ACTB) served as the reference gene (primer sequences are provided in Supplementary Table S1). The relative gene expression levels were calculated using the 2^−ΔΔCt^ method.

### 4.14. Statistical Analysis

Statistical analysis was performed using one-way analysis of variance (ANOVA) with Tukey’s post hoc multiple comparisons test in SPSS version 22 (IBM Corp., Armonk, NY, USA). This parametric test was chosen based on the assumptions of normality and homoscedasticity of variances across groups. Data are presented as mean ± standard error of the mean (SEM). Statistical significance was set at *p* < 0.05. Specific *p*-values are reported in the figure legends, with different letters above the columns indicating significant differences between groups (*p* < 0.05).

## 5. Conclusions

Our study demonstrates the potential of OFBP hydrolysate to combat age-related kidney decline by targeting ferroptosis. In vitro, OFBP treatment protected kidney tubular cells from ferroptosis and preserved mitochondrial health. In vivo, OFBP administration improved histological markers of aging kidneys and reduced markers of ferroptosis. Furthermore, OFBP modulated ferroptosis-regulatory molecules, suggesting its therapeutic potential. Further research is needed to explore the mechanisms of OFBP’s action and its efficacy in more complex models.

## Data Availability

All data supporting this study’s findings are available from the corresponding authors depending on reasonable request.

## Supplementary Materials

The following supporting information can be downloaded at: https://www.mdpi.com/article/10.3390/ijms25094668/s1.
