# Supplementary material for: Olive Flounder By-Product Prozyme2000P Hydrolysate Ameliorates Age-Related Kidney Decline by Inhibiting Ferroptosis
Source: Int J Mol Sci. 2024 Apr 25;25(9):4668. doi: 10.3390/ijms25094668 (PMC11083375; doi:10.3390/ijms25094668)
Supplement: Supplementary file 1 [file ijms-25-04668-s001.zip › ijms-2949711-supplementary.pdf]

Supplementary Materials

Supplementary Table S1. Primer sequence for qRT-PCR

Name	Organism	Forward sequence (5′ to 3′)	Reverse sequence (5′ to 3′)
Slc7a11	Mus musculus (Mouse)	CTT TGT TGC CCT CTC CTG CTT C	CAG AGG AGT GTG CTT GTG GAC A
Gpx4	Mus musculus (Mouse)	CCT CTG CTG CAA GAG CCT CCC	CTT ATC CAG GCA GAC CAT GTG C
Chac1	Mus musculus (Mouse)	TGA CCC TCC TTG AAG ACC GTG A	AGT GTC ATA GCC ACC AAG CAC G
Alox15	Mus musculus (Mouse)	GAC ACT TGG TGG CTG AGG TCT T	TCT CTG AGA TCA GGT CGC TCC T
Actb	Mus musculus (Mouse)	CAT TGC TGA CAG GAT GCA GAA GG	TGC TGG AAG GTG GAC AGT GAG G

Supplementary Figure S1. UPLC-Q-TOF MS/MS spectrum of mass produced OFBP

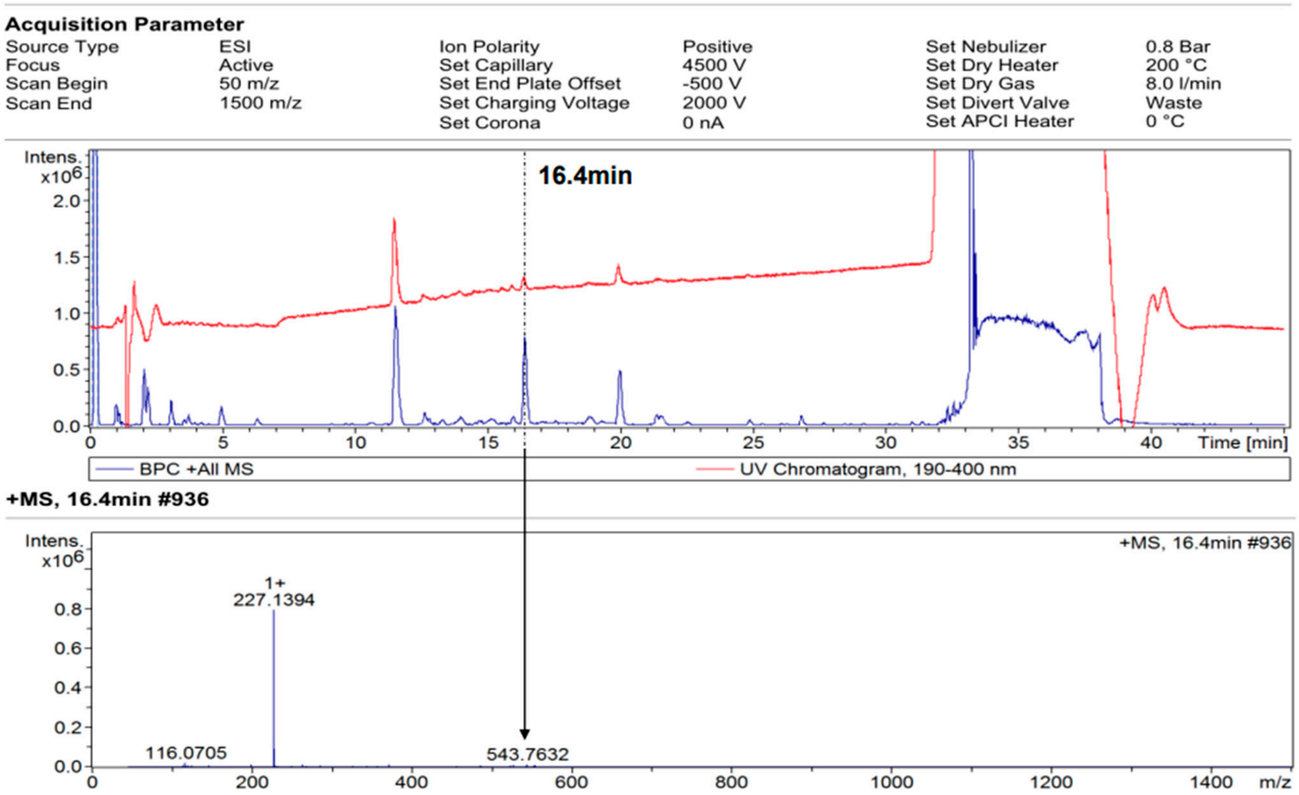

This figure show that GASGERGEVGPA purified from *P. olivaceus* was analyzed by liquid chromatography-tandem mass spectrometry (LC-MS/MS). The molecular mass and amino sequences. The LC-MS/MS spectrum of GASGERGEVGPA showed peaks at 543.7632^{2+} m/z.
